# Myeloid but not epithelial tissue factor exerts protective anti‐inflammatory effects in acid aspiration‐induced acute lung injury

**DOI:** 10.1111/jth.13737

**Published:** 2017-06-20

**Authors:** J. B. Kral‐Pointner, W. C. Schrottmaier, V. Horvath, H. Datler, L. Hell, C. Ay, B. Niederreiter, B. Jilma, J. A. Schmid, A. Assinger, N. Mackman, S. Knapp, G. Schabbauer

**Affiliations:** ^1^ Institute for Physiology Center for Physiology and Pharmacology Medical University of Vienna Vienna Austria; ^2^ Clinical Division of Hematology and Hemostaseology Department of Medicine I Medical University of Vienna Vienna Austria; ^3^ Division of Rheumatology Internal Medicine III Medical University of Vienna Vienna Austria; ^4^ Departments of Clinical Pharmacology Department of Medicine I Medical University of Vienna Vienna Austria; ^5^ Department for Vascular Biology and Thrombosis Research Center for Physiology and Pharmacology Medical University of Vienna Vienna Austria; ^6^ Division of Hematology/Oncology, Thrombosis and Hemostasis Program UNC McAllister Heart Institute University of North Carolina Chapel Hill NC USA; ^7^ CEMM Research Center for Molecular Medicine of the Austrian Academy of Sciences Vienna Austria; ^8^ Laboratory of Infection Biology Department of Medicine I Medical University of Vienna Vienna Austria

**Keywords:** acute lung injury, blood coagulation, inflammation, macrophages, tissue factor

## Abstract

**Essentials**

- Tissue factor (TF) represents a central link between hemostasis and inflammation.
- We studied the roles of myeloid and airway epithelial TF in acid‐caused acute lung injury (ALI).
- TF on myeloid cells displays a non‐coagulatory role regulating the inflammatory response in ALI.
- Airway epithelial TF contributes to hemostatic functions, but is dispensable in ALI pathogenesis.

**Summary:**

**Introduction**

Acute lung injury (ALI) is a life‐threatening condition characterized by damaged alveolar–capillary structures and activation of inflammatory and hemostatic processes. Tissue factor (TF) represents a crucial link between inflammation and coagulation, as inflammatory mediators induce myeloid TF expression, and TF initiates extrinsic coagulation.

**Objective**

As pulmonary inflammation stimulates TF expression and TF modulates immune responses, we aimed to elucidate its impact on ALI. In particular, we wanted to distinguish the contributions of TF expressed on airway epithelial cells and TF expressed on myeloid cells.

**Methods**

Mice with different cell type‐specific TF deficiency and wild‐type littermates were intratracheally treated with hydrochloric acid, and leukocyte recruitment, cytokine levels, thrombin–antithrombin (TAT) complexes and pulmonary protein‐rich infiltrates were analyzed.

**Results**

Our data demonstrate that a lack of epithelial TF did not influence acute responses, as bronchoalveolar neutrophil accumulation 8 h after ALI induction was unaltered. However, it led to mild, prolonged inflammation, as pulmonary leukocyte and erythrocyte numbers were still increased after 24 h, whereas those in wild‐type mice had returned to basal levels. In contrast, myeloid TF was primarily involved in regulating the acute phase of ALI without affecting local coagulation, as indicated by increased bronchoalveolar neutrophil infiltration, pulmonary interleukin‐6 levels, and edema formation, but equal TAT complex formation, 8 h after ALI induction. This augmented inflammatory response associated with myeloid TF deficiency was confirmed *in vitro*, as lipopolysaccharide‐stimulated TF‐deficient alveolar macrophages released increased levels of chemokine (C‐X‐C motif) ligand 1 and tumor necrosis factor‐α as compared with wild‐type macrophages.

**Conclusion**

We conclude that myeloid TF dampens inflammation in acid‐induced ALI.

## Introduction

Tissue factor (TF), also referred to as factor III, thromboplastin, or CD142, is a glycosylated integral transmembrane protein that initiates the extrinsic coagulation cascade. TF is constitutively expressed on the surface by various extravascular tissues, such as pulmonary epithelium. Upon injury, e.g. to the alveolar–endothelial barrier, extravascular TF is exposed to plasmatic coagulation factors, e.g. FVII, and initiates clot formation. The generated thrombin triggers fibrin formation, which stabilizes the primary platelet plug. Hence, TF is important for protecting vascular integrity, and thus preventing uncontrolled and life‐threatening blood loss 1, 2, 3. Upon infection or inflammation, TF expression is readily induced on leukocytes that are usually devoid of surface TF. These TF‐expressing leukocytes comprise members of the first line of defense, such as macrophages, but also circulating monocytes or neutrophils. Furthermore, blood vessel‐lining endothelial cells are able to synthesize TF 4, 5, 6. Stimuli triggering bloodborne TF expression during infection include pathogen‐associated molecular patterns, e.g. lipopolysaccharide (LPS) or lipoteichoic acid, and endogenous inflammatory cytokines, e.g. tumor necrosis factor (TNF)‐α and interleukin (IL)‐1β 7, 8. This process involves several transcription factors that are essential for immune functions, such as nuclear factor‐κB (NF‐κB), AP‐1, nuclear factor of activated T cells, and early growth response‐1 9, 10, 11, 12. Hence, the properties of TF are not strictly confined to hemostatic functions, but also promote host defense and inflammation 13. Therefore, reduced TF activity in mice leads to more severe bacteremia and increased mortality 14. However, uncontrolled coagulation cascade activation markedly contributes to disseminated intravascular coagulation. Correspondingly, reduced TF expression protects mice from systemic endotoxemia 15, indicating that finely balanced TF expression is essential for adequate immune responses during sepsis. Furthermore, TF inhibition prevents secondary lung injury in a baboon sepsis model 16. However, it remains incompletely understood how TF contributes to local infection and inflammation in organs such as the lung.

Acute lung injury (ALI) represents a multifactorial disease that can be induced by aspiration of non‐infectious acidic gastric contents as well as by pathogenic lung infections. The role of TFs in different pathologies of lung injury are still undefined. A recent study investigating low‐TF mice subjected to LPS‐induced ALI suggested that reduced TF expression exacerbates disease pathogenesis 17. Cell type‐specific TF deficiency in type I and type II lung epithelial cells worsens LPS‐induced ALI by increasing alveolar permeability and hemorrhage. In contrast, mice devoid of myeloid TF show no overt differences in the severity of lung damage 18. In the present study, we focused on cell type‐specific roles of TF in acid‐induced ALI. This clinically relevant mouse model mimics aspiration of gastric contents leading to lung complications (particularly in patients admitted to intensive care units), which accounts for 11% of all ALI cases in clinics 19, and is characterized by the release of endogenous alarm signals, damage‐associated molecular patterns (DAMPs), and simultaneous Toll‐like receptor activation 20, 21. The resulting inflammation is associated with endothelial cell activation, leukocyte and platelet influx, activation of coagulation, fibrin deposition, and, eventually, damage to the alveolar–capillary barrier 22. Notably, activation of the coagulation cascade plays a relevant role in disease progression 23.

As coagulatory and inflammatory processes affect the pathogenesis of ALI, we wanted to elucidate the impact of TF on these events during ALI. Thus, we endeavored to elucidate whether the presence of TF is required to protect the integrity of alveolar–capillary structures. Moreover, we aimed to define cell type‐specific functions of TF, and therefore performed acid‐induced ALI in mice with TF deficiency in either myeloid cells (monocytes, macrophages, and granulocytes) or alveolar type I and type II epithelial cells. Our data indicate that TF acts in a cell type‐specific manner, and that myeloid TF, in contrast to airway epithelial TF, dampens inflammatory responses during acid‐induced ALI.

## Materials and methods

### Mice

All experiments and animal studies were conducted according to institutional guidelines and were approved by the Animal Care and Use Committee of the Medical University of Vienna (BMWF‐66.009/0320‐II/3b/2013; BMWF‐66.009/0254‐ll/3b/2013).

All animal experimentation was littermate‐controlled. We used previously described LysM cre^+/−^TF^flox/flox^ (referred to as TF^∆mye^) 24, 25 or SPC cre^+/−^TF^flox/flox^ (referred to as TF^∆epi^) 18 mice on a C57BL/6J genetic background (backcrossed for six generations), and 8–12‐week‐old, sex‐matched littermates (control LysM cre^−/−^TF^flox/flox^ and SpcCre^−/−^TF^flox/flox^, referred as TF^+/+^ or wild‐type).

Direct PCR of lysed tissue was performed for genotyping. (Primers: TF forward, 5′‐ATGAGGAGCTGTGTTAAAGGGTCGCAGA‐3′; TF reverse, 5′‐TGCAGTAAATGCACGTGTCTGCCAT‐3′; Cre forward, 5′‐TCGCGATTATCTTCTATATCTTCAG‐3′; Cre reverse, 5′‐GCTCGACCAGTTTAGTTACCC‐3′.)

### Anesthesia

Mice were anesthetized with Ketaminol (50 mg kg^−1^) (Intervet International, Unterschleißheim, Germany) and Xylasol (5 mg kg^−1^) (Dr E. Gräub, Bern, Switzerland) or Forane (isoflurane; Abbott Laboratories, Queenborough, UK).

### ALI and administration of anti‐TF antibody

Sedated mice were intratracheally treated with 50 μL of 0.1 m hydrochloric acid (HCl) to induce ALI.

Wild‐type mice (C57BL/6J genetic background) were intraperitoneally injected once with monoclonal inhibitory rat anti‐mouse TF (1H1) (20 μg g^−1^) prior to ALI induction.

### Bronchoalveolar lavage fluid (BALF) and alveolar macrophage isolation and stimulation

For quantification of cytokines and leukocytes in the bronchoalveolar space, the trachea was exposed and punctured with a vein catheter, and the bronchoalveolar space was lavaged with 1 mL of phosphate‐buffered saline (PBS). Cells were pelleted for flow cytometry (600 × *g*, 10 min), and supernatants were used for ELISA.

Alveolar macrophages were isolated by repeating lavaging (10 times), and seeded into non‐tissue culture‐treated plates (2 h, 37 °C, and 5% CO_2_; RPMI medium (Sigma‐Aldrich, Vienna, Austria) dosed with 10% fetal bovine serum, 1% penicillin, streptomycin, and fungizone, and 1% l‐glutamine) before non‐adherent lymphocytes were discarded.

Alveolar macrophages were stimulated with 10 ng mL^−1^ or 100 ng mL^−1^ LPS (LPS‐EB ultrapure from *Escherichia coli* O111:B4; Invivogen, Toulouse, France) for the indicated times, and supernatant or cells with peqGOLD TriFast (VWR, Darmstadt, Germany) were harvested.

### Bone marrow‐derived macrophage (BMDM) isolation

Murine bone marrow was isolated and incubated with granulocyte–macrophage colony‐stimulating factor (GM‐CSF) (10 ng mL^−1^) for 1 week to generate BMDMs, which were then stimulated with LPS (10 ng mL^−1^) for the indicated times.

### Lung homogenization for ELISA and western blotting

Lungs were homogenized in 4 μL of PBS mg^–1^ with 5‐mm beads in a Precellys24 homogenizer (Bertin Technologies, Aix‐en‐Provence, France). Supernatants were mixed with Greenberger Lysis buffer and protease inhibitor (Roche Life Sciences, Basel, Switzerland), incubated (20 min on ice), and centrifuged (135 × *g*, 15 min, 4 °C), and the supernatant was used for ELISA.

For immunoblotting, lungs were homogenized in 10 μL of RIPA buffer mg^–1^, and supernatants were mixed with Laemmli buffer.

### RNA isolation and quantitative PCR (qPCR)

RNA was isolated from TriFast‐homogenized lungs or macrophages, transcribed into cDNA (High‐Capacity cDNA Reverse transcription Kit; Applied Biosystems, Carlsbad, CA, USA), and analyzed by qPCR with Fast SYBR Green Master Mix (Applied Biosystems, Carlsbad, CA, USA) on a StepOne Real‐Time PCR System (Applied Biosystems, Foster City, CA, USA). The results were evaluated according to Pfaffl 26 and expressed as fold control values. Primer sequences are supplied in Data S1.

### Western blotting

Proteins were separated by SDS‐PAGE (10% acrylamide gels), and semi‐dry‐blotted onto a poly(vinylidene difluoride) membrane. The fibrin β‐monomer 59D8 and anti‐β‐actin (Sigma‐Aldrich) and horseradish peroxidase‐linked secondary antibodies were used. Immunoblots were developed with SuperSignal West Femto Maximum Sensitivity Substrate (Thermo Fisher Scientific, Waltham, MA, USA), imaged with FluorChem HD2 Chemiluminesence Imager (Alpha Innotech, San Leandro, CA, USA), and densitometrically analyzed with imagej 1.47v. Bands were normalized to β‐actin.

### Flow cytometry

BALF cells were incubated (20 min) with anti‐CD45–PerCP, anti‐F4/80–fluorescein isothiocyanate, anti‐Ly6G/Ly6C–phycoerythrin, and anti‐Ter119–allophycocyanin (1 : 80; BioLegend, London, UK), fixed with 1% formaldehyde, and measured by flow cytometry (BD Accuri flow cytometer with BD accuric6, and flowjo 9 [Treestar, Ashland, OR, USA]). The following gating strategy was applied. CD45^+^ cells (leukocytes) were analyzed for F4/80 positivity (macrophages). Ly6C^+^ macrophages were termed inflammatory macrophages. CD45^+^ F4/80^−^ cells were analyzed for Ly6G expression (neutrophils).

Total cell counts of BALF were calculated by multiplying the events by

$$\begin{array}{cc} & \left(\frac{\text{Final dilution for flowcytometer}}{\text{Measured volume}}\times \frac{\text{Resupensionvolume}}{\text{Stained volume}}\right)\\ & \times \frac{\text{Injected PBS}}{\text{Recollected PBS}} \end{array}$$

### Histologic analysis

The left lung was extracted, fixed in 4% formaldehyde, and embedded in paraffin. Three‐micrometer lung sections were stained with anti‐mouse GR‐1 or TF antibody, and counterstained with hematoxylin or with hematoxylin and eosin (Carl Roth, Karlsruhe, Germany). Images were obtained with an Axio Imager.Z1 microscope (Carl Zeiss, Vienna, Austria).

### TF activity assay

TF activity was determined as previously described 27. Briefly, BALF was diluted 1 : 6 with Hanks’ balanced salt solution with 0.1% bovine serum albumin (HBSA), and microvesicles were pelleted (18 000 × *g*, 20 min, 4 °C), washed, and incubated with anti‐mouse TF (1H1) (100 μg mL^−1^) or control IgG (15 min) before addition of FVII (4.88 nm), FX (150 nm), and CaCl_2_ (10 mm). The reaction was stopped by addition of EDTA HBSA buffer before addition of chromogenic substrate and absorbance measurement. TF‐dependent activated FX (FXa) generation was determined by subtracting FXa levels generated in the presence of TF antibody from the levels in IgG control samples.

### ELISA

ELISAs were performed according to manufacturer's protocols. Myeloperoxidase (MPO), IL‐33 and IL‐6, and chemokine (C‐X‐C‐motif) ligand‐1 (KC), TNF‐α and thrombin–antithrombin (TAT) ELISAs were purchased from eBioscience (San Diego, USA), R&D Systems (Minneapolis, MN, USA) and Siemens Healthcare Diagnostics (Vienna, Austria), respectively. Absorbance was measured at 450 nm with an EL808 Ultra Microplate Reader (Bio‐Tek Instruments, Winooski, VT, USA).

### Bicinchoninic acid (BCA) assay

The protein concentration in BALF was determined with a Pierce BCA Protein Assay Kit (Thermo Fisher Scientific, Waltham, MA, USA) according to the manufacturer's guidelines.

### Edema formation

Murine lungs were extracted, weighed, dried at 55 °C for 48 h, and weighed again. The ratio between wet weight and dry weight was calculated.

### Statistical analysis

Data were depicted as bar diagrams showing the mean *±* standard error of the mean, as box plots indicating the median, the first and third quartiles, and the minimum and maximum, or as scatter plots with indicated means. The results were evaluated with graphpad prism 5 (GraphPad, San Diego, CA, USA) by one‐way‐anova with Dunn's multiple comparisons test, two‐way anova with Sidak's multiple comparisons test, unpaired, two‐tailed *t*‐tests, or the Mann–Whitney test, if the samples did not pass the Kolmogorov–Smirnov test for normal distribution. A *P*‐value of < 0.05 was considered to be significant.

## Results

### TF expression during acid‐induced ALI

**Figure 1:**
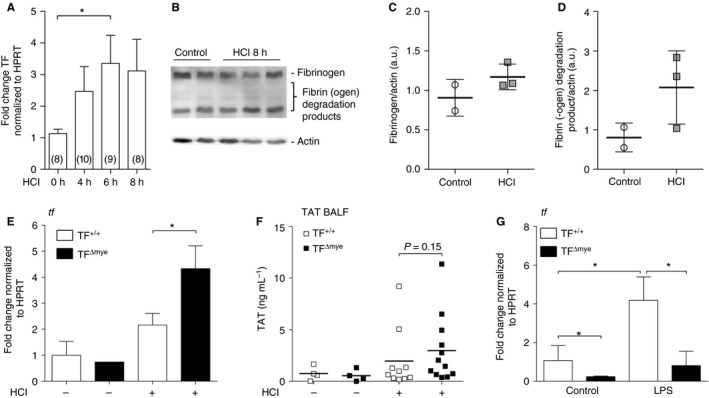
Tissue factor (TF) expression and activation of coagulation during acute lung injury (ALI). (A) TF mRNA expression in lungs of wild‐type mice after HCl‐induced ALI was determined by qPCR at the indicated time points. Data show fold control (0 h) values normalized to hypoxanthine guanine phosphoribosyl transferase (HPRT); numbers are shown in parentheses. (B) Western blot analysis for fibrin‐β of homogenized lung samples 8 h after HCl treatment (*n* = 3) and of controls (*n* = 2); actin was used as a loading control. (C, D) Western blot densitometric analysis of (C) fibrinogen and (D) the major fibrin(ogen) degradation product. (E) TF mRNA expression in total lung tissue of control (*n* _TF__+/+_ = 2; *n* _TF__Δmye_ = 1) and HCl‐treated (*n* _TF__+/+_ = 9; *n* _TF__Δmye_ = 4) myeloid TF‐deficient (TF ^Δmye^) or wild‐type (TF ^+/+^) mice was analyzed by q‐PCR 8 h after treatment. (F) Thrombin–antithrombin (TAT) complexes in the bronchoalveolar lavage fluid (BALF) of control (*n* = 4) and HCl‐treated (*n* _TF__+/+_ = 10; *n* _TF__Δmye_ = 12) wild‐type or myeloid TF‐deficient mice 8 h after treatment. (G) TF mRNA expression of alveolar macrophages, isolated from lungs of myeloid TF knockout mice and wild‐type littermates and stimulated with lipopolysaccharide (LPS) (100 ng mL^−1^) *in vitro* for 3 h; *n* = 5. For statistical analysis, one‐way‐anova with Dunn's multiple comparisons test (A), unpaired Student's *t*‐tests (E, F) or the Mann–Whitney test (G) were performed; *P < 0.05. Littermate‐controlled experiments were performed. a.u., arbitrary units.

Activation of coagulation is an integral step in the pathology of ALI. We established a mouse model of acid‐induced ALI by intratracheal administration of HCl. Lung TF mRNA expression was increased within the first 8 h of ALI, which represents the most critical time in the pathology (Fig. 1A), whereas we could not detect simultaneous accumulation of fibrin β‐monomers in lungs (Fig. 1B–D). This indicates rather modest activation of coagulation during acid‐induced ALI. To investigate the role of TF in acid‐induced ALI, we used mice with a cell type‐specific TF deficiency (Fig. S1) either in myeloid cells (TF^Δmye^) or in lung epithelial cells (TF^Δepi^) in littermate‐controlled experiments. We found a significant increase in TF mRNA expression in TF^Δmye^ mice during ALI, which was significantly more pronounced than in wild‐type mice (Fig. 1E). This indicates that non‐myeloid cells upregulate TF during ALI. As fibrin generation was only moderately affected during ALI, we used thrombin, the protease‐cleaving fibrinogen, as a functional readout for coagulation activation. Formed thrombin becomes rapidly inactivated via binding of antithrombin III, leading to the formation of TAT complexes. However, we found only modest TAT complex upregulation in BALF 8 h after ALI, perhaps because of low FVII and FX BALF levels. TAT complex formation only tends to be increased in TF^Δmye^ mice (Fig. 1F). *In vitro*, we confirmed TF deletion at the mRNA level in alveolar macrophages with and without LPS stimulation (Fig. 1G).

### TF on myeloid cells negatively regulates leukocyte recruitment during acid‐induced ALI

**Figure 2:**
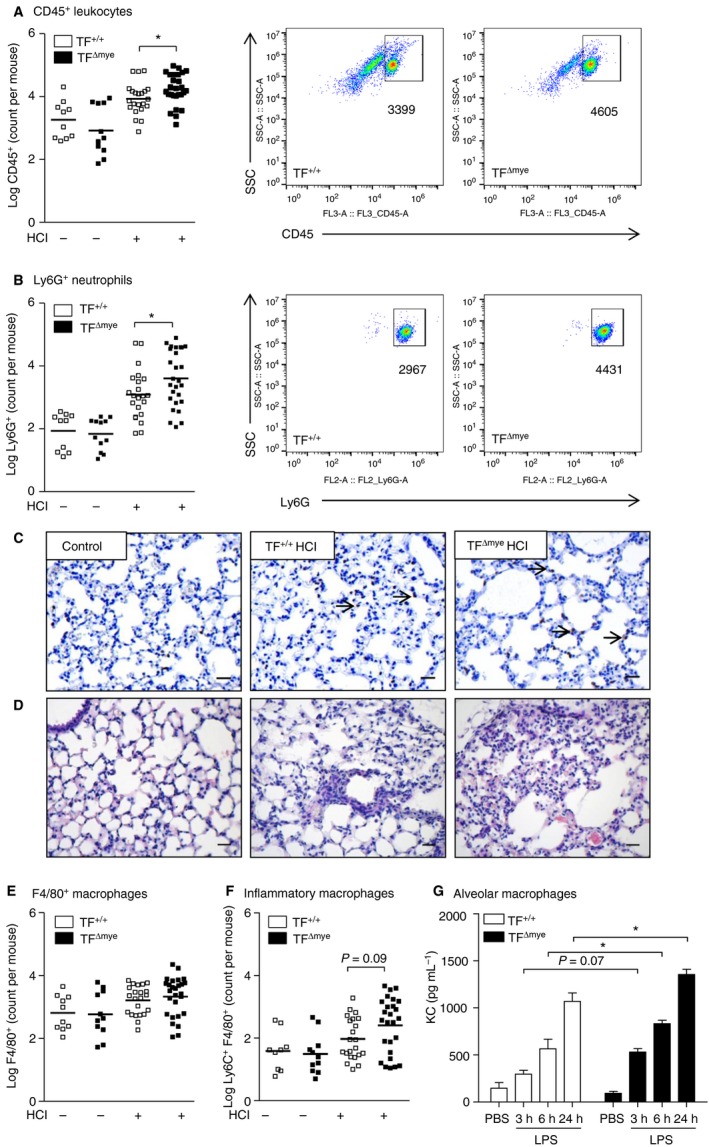
The effect of myeloid tissue factor (TF) on pulmonary leukocyte recruitment during acute lung injury (ALI). (A, B, E, F) Flow cytometric analysis of leukocytes in the bronchoalveolar lavage fluid of control mice (*n* _TF__+/+_ = 10; *n* _TF__Δmye_ = 11) and HCl‐treated (*n* _TF__+/+_ = 22; *n* _TF__Δmye_ = 27) myeloid TF‐deficient mice or wild‐type mice 8 h after ALI. (A) CD45^+^ leukocytes. (B) CD45^+^ Ly6G^+^ F4/80^−^ neutrophils. (E) CD45^+^ F4/80^+^ macrophages. (F) CD45^+^ Ly6C^+^ F4/80^+^ inflammatory macrophages (*n*_control_ _TF__+/+_ = 9; *n*_control_ _TF__Δmye_ = 11; *n* _HC__l_ _TF__+/+_ = 21; *n* _HC__l_ _TF__Δmye_ = 26). (A, B) Representative blots of HCl‐treated mice. (C,D) Lung sections of myeloid TF‐deficient mice or wild‐type mice 8 h after ALI induction stained for GR‐1 and hematoxylin (C) or hematoxylin and eosin (D). Scale bar: 25 μm. Magnification: × 200. Arrows indicate neutrophils. (G) Alveolar macrophages were stimulated with lipopolysaccharide (LPS) for the indicated time points *in vitro*, and released chemokine (C‐X‐C‐motif) ligand‐1 (KC) was measured by ELISA;* n* = 3. For statistical analysis (A–F), unpaired Student's *t*‐tests and (G) two‐way anova with Sidak's multiple comparison were performed; *P < 0.05. Littermate‐controlled experiments were performed. PBS, phosphate‐buffered saline.

One of the most prominent features of ALI is leukocyte influx, in particular of neutrophils, into the bronchoalveolar compartment. As we observed only a minor impact of TF induction on coagulation, we investigated whether upregulated TF could influence inflammation during ALI. Acid‐induced ALI led to significantly enhanced leukocyte extravasation in TF^Δmye^ mice as compared with wild‐type littermates after 8 h, as evidenced by increased CD45^+^ leukocyte numbers (Fig. 2A). We further identified CD45^+^ F4/80^−^ Ly6G^+^ cells, representing neutrophils, as strongly contributing to increased cellular influx. Significantly more neutrophils migrated into the bronchoalveolar compartment in TF^Δmye^ mice 8 h after acid‐induced ALI (Fig. 2B). To verify that the majority of neutrophils transmigrated through the interstitial barrier, we stained lung sections for the neutrophil marker GR‐1 (brown staining, arrows), which recognizes both neutrophils and, to a lesser extent, activated macrophages. We observed that most of the neutrophils were present within the alveoli, and that only a subfraction were present in interstitial layers (Fig. 2C). Furthermore, leukocyte accumulation and lung damage in TF^Δmye^ mice were slightly increased as compared with wild‐type mice (Fig 2D). In contrast, flow cytometric analysis of macrophage subsets characterized by either F4/80^+^ (Fig. 2E; Fig. S2A) or F4/80^+^ CD11c^+^ (Fig. S2B) revealed no difference between TF^Δmye^ mice and wild‐type littermates. Only inflammatory macrophages (Fig. 2F), characterized by F4/80^+^ and Ly6C^+^ 28, 29, were marginally, but not significantly (*P* = 0.09), increased in number. To analyze the specificity of this phenomenon, we applied a leukocyte recruitment model to TF^Δmye^ and wild‐type mice. To this end, we induced thioglycollate‐elicited sterile inflammation, and analyzed leukocyte influx in an early phase (4 h) and a late phase (72 h). However, no differences in infiltrating leukocytes, macrophages or neutrophils were observed, perhaps because of the strong inflammatory stimulus (Fig. S3A–D). *In vitro* analysis of alveolar macrophages revealed that, in response to LPS, TF deficiency increased the release of the chemokine KC, which primarily attracts neutrophils (Fig. 2G). These data may indicate that myeloid TF plays a lung‐specific role in regulating leukocyte alveolar trafficking.

### TF dampens the inflammatory response in acid‐induced ALI

**Figure 3:**
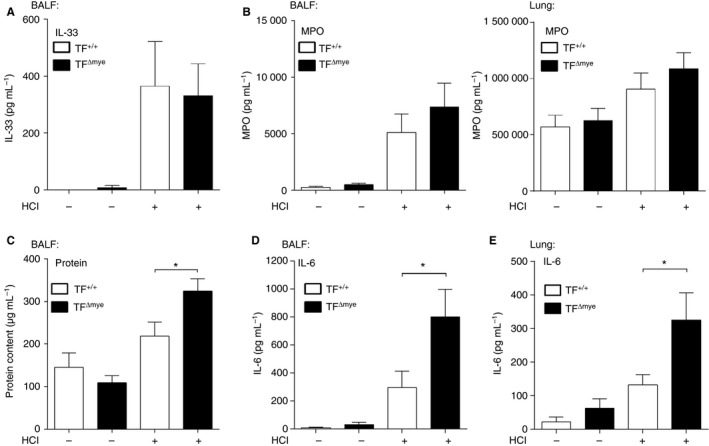
Increased inflammation in myeloid tissue factor (TF)‐deficient mice in response to acid‐induced acute lung injury (ALI). (A–D) Interleukin (IL)‐33 (A) (*n*_control_ _TF__+/+_ = 6, *n*_control_ _TF__Δmye_ = 5, *n* _HC__l_ _TF__+/+_ = 7, *n* _HC__l_ _TF__Δmye_ = 8), myeloperoxidase (MPO) (B) (bronchoalveolar lavage fluid [BALF] – *n*_control_ _TF__+/+_ = 6, *n*_control_ _TF__Δmye_ = 4, *n* _HC__l_ _TF__+/+_ = 11, *n* _HC__l_ _TF__Δmye_ = 12; lung – *n*_control_ _TF__+/+_ = 5, *n*_control_ _TF__Δmye_ = 5, *n* _HC__l_ _TF__+/+_ = 14, *n* _HC__l_ _TF__Δmye_ = 14) and IL‐6 (D) (*n*_control_ _TF__+/+_ = 6, *n*_control_ _TF__Δmye_ = 5, *n* _HC__l_ _TF__+/+_ = 10, *n* _HC__l_ _TF__Δmye_ = 12) were measured in BALF by ELISA. (C) The protein content in BALF was measured with a bicinchoninic acid protein assay (*n*_control_ _TF__+/+_ = 3, *n*_control_ _TF__Δmye_ = 3, *n* _HC__l_ _TF__+/+_ = 7, *n* _HC__l_ _TF__Δmye_ = 10). (E) IL‐6 levels in total lung tissue were determined by ELISA (*n*_control_ _TF__+/+_ = 7, *n*_control_ _TF__Δmye_ = 8; *n* _HC__l_ _TF__+/+_ = 11, *n* _HC__l_ _TF__Δmye_ = 13). Parameters were analyzed 8 h after HCl treatment. For statistical analysis, unpaired Student's *t*‐tests (A–E) were performed; *P < 0.05. Littermate‐controlled experiments were performed.

Another hallmark of acid‐induced ALI is the induction of an inflammatory response, which is usually triggered by endogenous alarmins or DAMPs, owing to cellular damage. DAMPs include nuclear components such as histones or high‐mobility group box 1, but also IL‐33 30. We observed a strong increase in the release of IL‐33 into the bronchoalveolar compartment in response to ALI, but no differences between TF^Δmye^ and wild‐type littermate mice (Fig. 3A). Although granulocyte numbers in TF^Δmye^ mice (Fig. 2B) were markedly increased, we found only a slight trend for increased MPO levels in BALF and lung homogenates in TF^Δmye^ mice (Fig. 3B). However, analysis of total protein in BALF revealed an increased protein content in TF^Δmye^ mice subjected to acid‐induced ALI as compared with wild‐type mice (Fig. 3C). This finding points towards increased vascular leakage and loss of alveolar–capillary barrier function in the lungs of TF^Δmye^ mice. Next, we measured the level of pulmonary IL‐6 of acid‐treated mice, which we considered to be the most robust marker for inflammation in this rather mild inflammatory model. We observed significantly increased IL‐6 production and release in BALF and total lung after acid‐induced ALI in TF^Δmye^ mice as compared with wild‐type mice (Fig. 3D,E). However, pulmonary IL‐1β and IL‐12 p40 levels were unaltered (Fig. S4A,B).

### TF dampens the inflammatory response of macrophages *in vitro*

**Figure 4:**
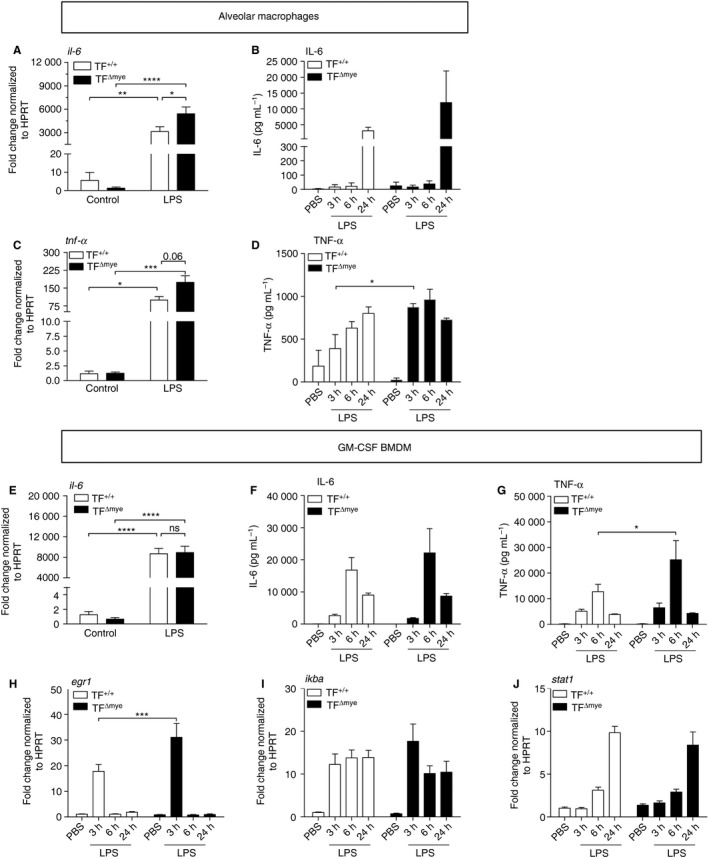
Myeloid tissue factor (TF) deficiency promotes a proinflammatory phenotype of alveolar and bone marrow‐derived macrophages (BMDMs). (A–D) Alveolar macrophages of TF ^Δmye^ and TF ^+/+^ mice were isolated and stimulated with lipopolysaccharide (LPS) (10 ng mL^−1^) or vehicle for the indicated time points. Interleukin (IL)‐6 (A) (*n* = 5) and tumor necrosis factor (TNF)‐α (C) (*n* = 3) mRNA expression in isolated alveolar macrophages, stimulated with LPS or vehicle for 3 h were determined by qPCR. Data are depicted as fold TF ^+/+^ control. (B, D) IL‐6 (B) and TNF‐α (D) protein levels in supernatants of alveolar macrophages were measured by ELISA at the indicated time points after LPS stimulation; *n* = 3. (E–J) Bone marrow cells of TF^Δmye^ and TF^+/+^ mice were differentiated towards macrophages by granulocyte–macrophage colony‐stimulating factor (GM‐CSF) (10 ng mL^−1^) and stimulated with LPS (10 ng mL^−1^) or vehicle at the indicated time points. (E) IL‐6 mRNA (*n*_control TF+/+_ = 5; all others *n* = 6) expression 3 h after stimulation was quantified by qPCR, and the data are depicted as fold TF^+/+^ control. (F) IL‐6 (*n*_6 h_ = 4; all others *n* = 6). (G) TNF‐α (*n* = 6) levels in supernatants were determined by ELISA. (H–J) EGR1 (H) (*n* = 5–6), IKBα (I) (*n* = 5–6) and STAT1 (J) (*n* = 5–6) were measured by qPCR, and are depicted as fold TF^+/+^ control. For statistical analysis, two‐way anova with Sidak's multiple comparison was performed; *P < 0.05, **P < 0.01, ***P < 0.001, ****P < 0.0001. Littermate‐controlled experiments were performed. HPRT, hypoxanthine guanine phosphoribosyl transferase; NS, not significant; PBS, phosphate‐buffered saline.

To determine the possible source of increased IL‐6 levels in HCl‐treated TF^Δmye^ mice and the impact of myeloid TF on inflammatory responses of macrophages, we isolated alveolar macrophages from naive TF^Δmye^ mice and wild‐type littermates. Analysis of IL‐6 at the mRNA and protein levels revealed a significant increase in IL‐6 mRNA 3 h after LPS stimulation (Fig. 4A,B). Moreover, TNF‐α mRNA and protein levels were significantly increased 3 h after LPS treatment (Fig. 4C,D). As alveolar macrophages are very limited in number, and their development depends on GM‐CSF 31, we also used GM‐CSF‐induced BMDMs. LPS‐stimulated TF‐deficient BMDMs expressed similar levels of IL‐6 mRNA and protein (Fig. 4E,F); however, TNF‐α secretion was significantly increased (Fig. 4G). To identify potential signaling pathways affected by TF, we analyzed the expression of EGR1, IκBα, and STAT1. We found that TF significantly reduced the expression of EGR1 1 h after LPS stimulation, whereas IκBα and STAT1 were similarly expressed (Fig. 4H–J), indicating that the mitogen‐activated kinase pathway was primarily affected, rather than the interferon and NF‐κB pathways.

As TF activity leads to activated FVII (FVIIa) and thrombin generation, we examined the effects of 3 h of human FVIIa and thrombin stimulation on wild‐type BMDMs. We found that concomitant stimulation with LPS and thrombin significantly reduced the expression of IL‐6 and tended to decrease the expression of TNF‐α and IκBα (Fig. S5A–C). Similarly, FVIIa showed a tendency to reduce IL‐6 levels and, to a lesser extent, TNF‐α and IκBα mRNA levels (Fig. S5D–F). However, *in vivo*, other mechanisms, such as the intrinsic coagulation cascade, can also contribute to thrombin generation. Moreover, TF can directly signal and thereby modulate macrophage functions.

These results indicate that the differential expression of proinflammatory cytokines during ALI is probably dependent on TF surface expression on innate immune cells.

### TF on epithelial cells does not contribute to tissue inflammation 8 h after acid‐induced ALI

**Figure 5:**
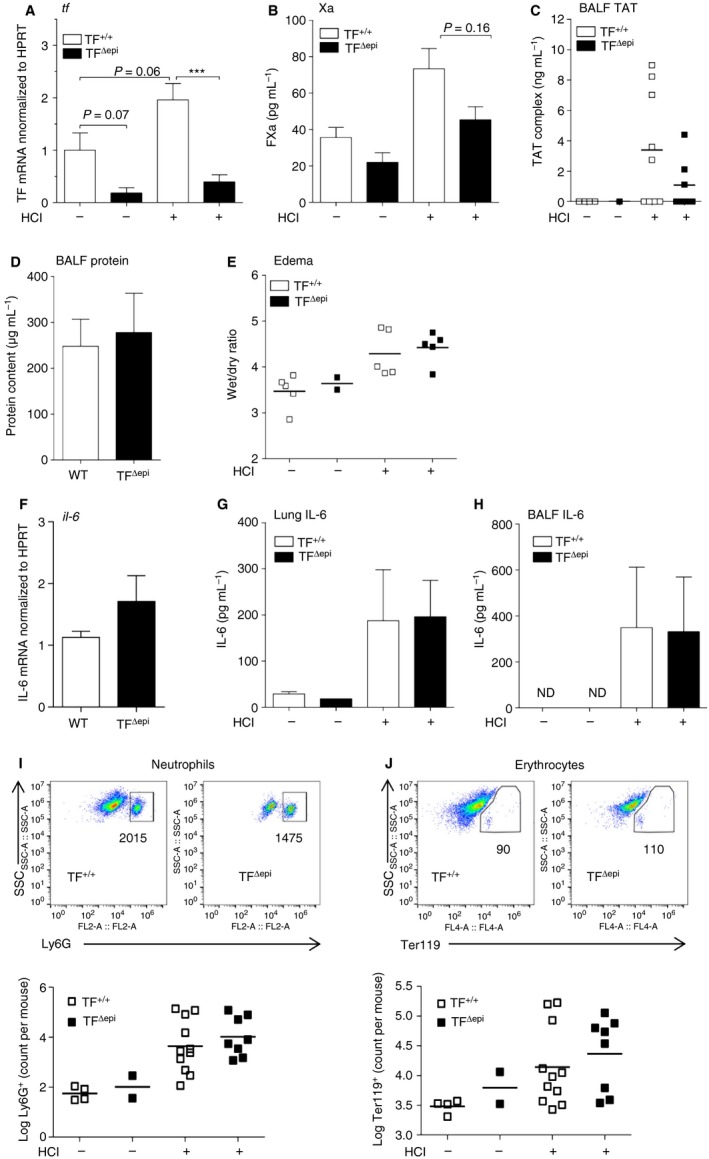
Epithelial tissue factor (TF) plays a minor role in acid‐induced acute lung injury (ALI). Epithelial TF‐deficient and wild‐type (WT) mice were treated with HCl for 8 h. (A) TF mRNA expression of lung tissue was determined by qPCR, and is depicted as fold wild‐type control (*n*_control_ _TF__+/+_ = 5; *n*_control_ _TF__Δmye_ = 3; *n* _HC__l_ _TF__+/+_ = 9; *n* _HC__l_ _TF__Δmye_ = 10). (B, C) Activated factor X (FXa) generation (B) (*n*_control_ _TF__+/+_ = 4; *n*_control_ _TF__Δmye_ = 2; *n* _HC__l_ _TF__+/+_ = 6; *n* _HC__l_ _TF__Δmye_ = 4) and thrombin–antithrombin (TAT) complex (C) were measured in bronchoalveolar lavage fluid (BALF) (*n*_control_ _TF__+/+_ = 4; *n*_control_ _TF__Δmye_ = 1; *n* _HC__l_ _TF__+/+_ = 9; *n* _HC__l_ _TF__Δmye_ = 7). (D) Protein content in BALF samples (*n*_control_ _TF__+/+_ = 4; *n*_control_ _TF__Δmye_ = 2; *n* _HC__l_ _TF__+/+_ = 10; *n* _HC__l_ _TF__Δmye_ = 6). (E) Lung edema was determined by measuring the weight ratio of wet and dried whole lung tissue (*n*_control_ _TF__Δmye_ = 2; all others *n* = 5) (F–H) IL‐6 mRNA expression (F) (*n*_control_ = 4; *n* _HC__l_ _TF__+/+_ = 5; *n* _HC__l_ _TF__Δmye_ = 8) and IL‐6 protein levels in (G) total lung tissue (*n*_control_ _TF__Δmye_ = 1; all others *n* = 4) and (H) BALF (*n*_control_ _TF__+/+_ = 4; *n*_control_ _TF__Δmye_ = 1; *n* _HC__l_ _TF__+/+_ = 6; *n* _HC__l_ _TF__Δmye_ = 3). (I, J) Flow cytometric analysis of (I) neutrophils (CD45^+^ Ly6G^+^ F4/80^−^) and (J) erythrocytes (Ter119^+^) in the BALF of epithelial TF‐deficient and WT mice (*n*_control_ _TF__+/+_ = 4; *n*_control_ _TF__Δmye_ = 2; *n* _HC__l_ _TF__+/+_ = 11; *n* _HC__l_ _TF__Δmye_ = 8). For statistical analysis, the Mann–Whitney test was performed (A, B); ***P < 0.001. Littermate‐controlled experiments were performed. HPRT, hypoxanthine guanine phosphoribosyl transferase; ND, not detectable.

To determine the contribution of lung epithelial TF to the pathophysiology of acid‐induced ALI, we used TF^Δepi^ mice. We used immunostaining to confirm that there was appropriate TF deficiency in the lungs of TF^Δepi^ mice 18 (Fig. S1B). Analysis of TF mRNA expression in whole lung tissue during ALI revealed a trend for increased TF expression 8 h after acid instillation in wild‐type mice. In contrast, TF mRNA levels were strongly reduced in TF^Δepi^ mice (Fig. 5A), indicating that epithelial cells express the majority of lung TF mRNA during ALI. The SPC cre efficiently targets TF expression in most of the lung epithelial cells 32. Moreover, upon LPS‐induced ALI, lung epithelial cells represent the major TF‐expressing cell type responsible for protection of alveolar–capillary integrity 18. Hence, we wanted to determine whether this is also the case during acid‐induced ALI. Measurement of TF activity revealed slightly increased FXa generation and TAT complex formation 8 h after disease induction in wild‐type littermates. In comparison, TF^Δepi^ mice showed a trend for reduced FXa generation but no differences in TAT complex formation (Fig. 5B,C). Next, we tested endothelial and epithelial barrier function by measuring BALF protein levels. Surprisingly, we did not observe significant differences in BALF protein levels and edema formation between acid‐treated TF^Δepi^ mice and wild‐type mice (Fig. 5D,E). Similarly, we did not observe alterations in erythrocyte levels in BALF (Fig. 5J). To test the inflammatory response, we measured pulmonary IL‐6 mRNA expression and IL‐6 protein levels in BALF and lung tissue, and pulmonary neutrophil transmigration, but did not detect any differences between acid‐treated TF^Δepi^ mice and wild‐type littermates (Fig. 5F–I).

### Restoration of tissue integrity is disturbed at late stages of acid‐induced ALI in TF^Δepi^ mice but not in TF^Δmye^ mice

**Figure 6:**
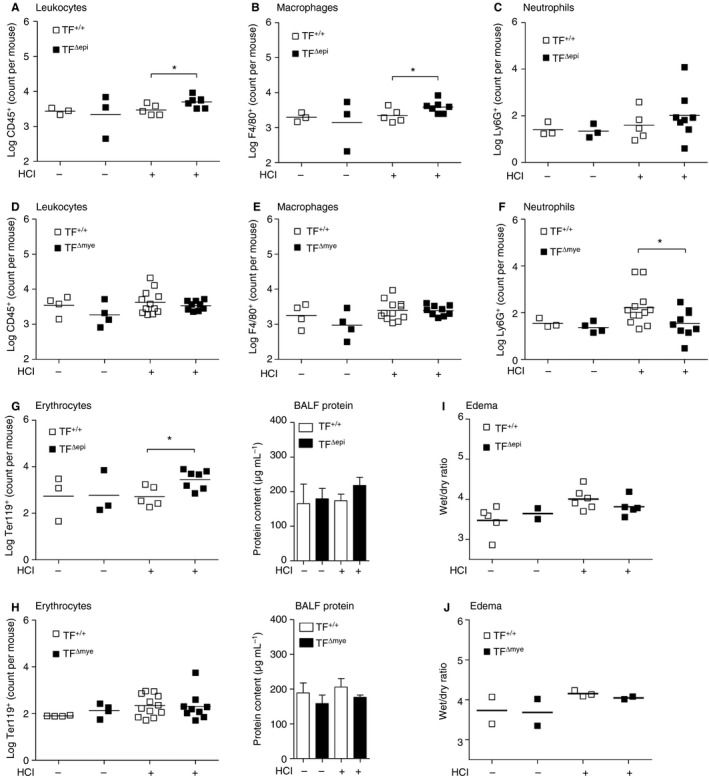
Leukocyte extravasation into the lung 24 h after HCl‐induced acute lung injury (ALI) in epithelial and myeloid tissue factor (TF)‐deficient mice. The presence of (A, D) leukocytes (CD45^+^), (B, E) macrophages (CD45^+^ F4/80^+^), (C, F) neutrophils (CD45^+^ Ly6G^+^ F4/80^−^) and (G, H) erythrocytes (Ter119^+^) in the bronchoalveolar lavage fluid (BALF) was determined by flow cytometry. (I, J) BALF protein was measured with a bicinchoninic acid assay. (A–C, G) *n*_control_ = 2–3; *n*_HCl TF+/+_ = 5; *n*_HCl TFΔepi_ = 7–8. (D–F, H) *n*_control_ = 3–4; *n*_HCl TF+/+_ = 12; *n*_HCl TFΔmye_ = 9. (I) *n*_control TF+/+_ = 5; *n*_control TF+/+_ = 2; *n*_HCl TF+/+_ = 6; *n*_HCl TFΔepi_ = 5. (J) *n* = 2–3. For statistical analysis, unpaired Student's *t*‐tests were performed; *P < 0.05. Littermate‐controlled experiments were performed.

The current literature on TF in ALI pathogenesis is focused on early responses in disease progression. Therefore, we aimed to elucidate the effects of TF deletion at late stages of ALI. Twenty‐four hours after acid treatment, the initial injury and inflammation in wild‐type mice had resolved. Extravasation of leukocytes, including neutrophils and macrophages, as well as erythrocytes and protein infiltrates, returned to baseline levels (Fig. 6A–H). Surprisingly, we observed increased leukocyte counts in the bronchoalveolar compartment in TF^Δepi^ mice as compared with wild‐type littermates at this time point, suggesting that TF^Δepi^ mice were unable to completely resolve the mild acid‐induced injury (Fig. 6A). Notably, most of this increase could be attributed to macrophages, and not to neutrophils (Fig. 6B,C). In contrast, TF^Δmye^ mice did not show any overt pulmonary inflammation 24 h after ALI induction, as indicated by baseline levels of leukocytes, macrophages and neutrophils in BALF (Fig. 6D–F). Similarly, we found significantly more erythrocytes in HCl‐treated TF^Δepi^ mice and a trend for more protein in the BALF (Fig. 6G), whereas TF^Δmye^ mice and wild‐type mice did not show increased levels in comparison with untreated mice (Fig. 6H). Edema formation was still slightly increased in all four HCl‐treated groups, but no differences between genotypes were observed (Fig. 6I,J).

### Systemic TF targeting by antibody does not affect inflammation but increases the risk of bleeding during acid‐induced ALI

**Figure 7:**
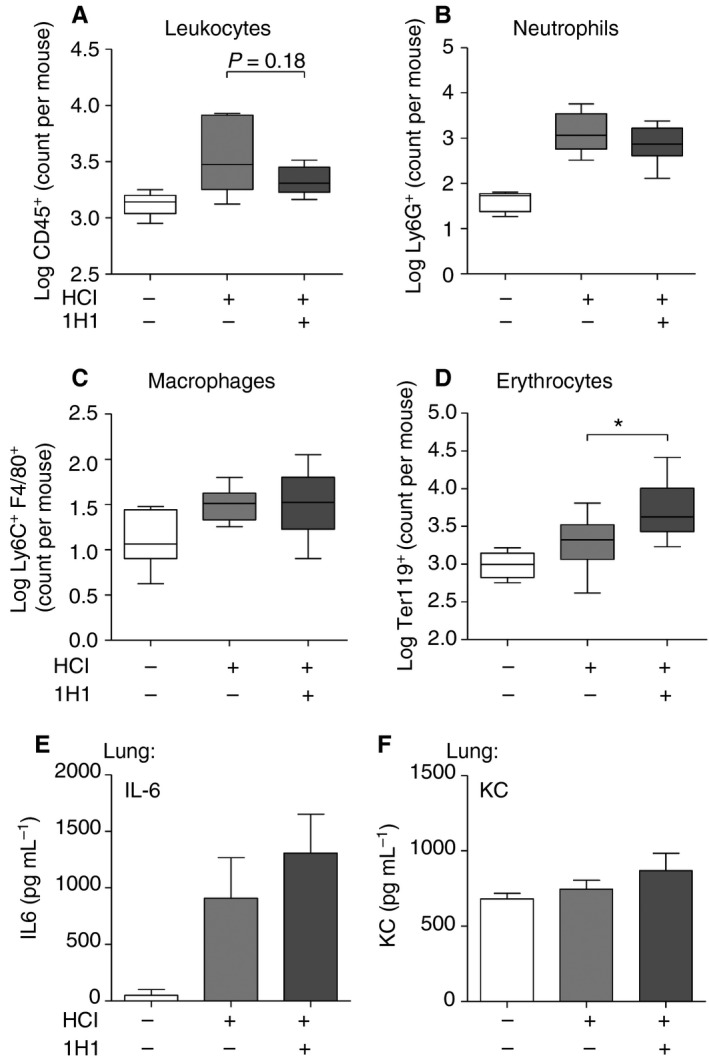
Systemic tissue factor (TF) inhibition in acid‐induced acute lung injury (ALI). Wild‐type mice were intraperitoneally injected with anti‐TF blocking antibody (1H1) or vehicle prior to HCl treatment, and parameters were analyzed 8 h after HCl treatment. (A–D) Extravasation of (A) leukocytes (CD45^+^), (B) neutrophils (CD45^+^ Ly6G^+^ F4/80^−^), (C) macrophages (CD45^+^ F4/80^+^) and (D) erythrocytes (Ter119^+^) in the bronchoalveolar lavage fluid (BALF) was determined by flow cytometry. (E, F) Interleukin (IL)‐6 (E) and chemokine (C‐X‐C‐motif) ligand‐1 (KC) (F) levels in BALF were determined by ELISA. (A) *n*_control_ _WT_ = 5; *n* _HC__l_ _WT_ = 12; *n* _HC__l 1H1_ = 9. (B) *n*_control_ _WT_ = 5; *n* _HC__l_ _WT_ = 8; *n* _HC__l 1H1_ = 8. (C) *n*_control_ _WT_ = 7; *n* _HC__l_ _WT_ = 8; *n* _HC__l 1H1_ = 8. (D) *n*_control_ _WT_ = 7; *n* _HC__l_ _WT_ = 10; *n* _HC__l 1H1_ = 10. (E, F) *n*_control_ _WT_ = 3; *n* _HC__l_ _WT_ = 6; *n* _HC__l 1H1_ = 6. For statistical analysis, unpaired Student's *t*‐tests were performed; *P < 0.05. Littermate‐controlled experiments were performed.

To elucidate a possible systemic role of TF in the progression of acid‐induced ALI, we administered the TF‐blocking antibody 1H1 intraperitoneally in C57BL/6J littermates 33. We observed no adverse events such as fatal bleeding during the 8‐h observation period after intratracheal acid instillation. When we analyzed ALI‐induced leukocyte influx in BALF, we found no differences for CD45^+^ leukocytes, Ly6G^+^ neutrophils, or F4/80^+^ macrophages (Fig. 7A–C). In contrast to what was seen in cell type‐specific TF‐deficient mice, systemic blockade of TF led to a significant increase in erythrocyte numbers in BALF (Fig. 7D), indicating that TF is required to maintain bronchoalveolar barrier function. Accordingly, we did not observe gross changes in IL‐6 (Fig. 7E) or KC (Fig. 7F) levels.

## Discussion

TF and the extrinsic coagulation cascade are intricately involved in the disease mechanisms underlying ALI. For instance, exacerbated fibrin deposition is detrimental to the function of the alveolar–capillary system by causing barrier dysfunction and hampering gas exchange 34. We found that myeloid TF deficiency exacerbated the pathology of acid‐induced ALI. Surprisingly, TF deficiency of lung epithelial cells resulted in an unusually long‐lasting (24 h) disease pathology of acid‐induced ALI, accompanied by modest intra‐alveolar hemorrhage. This is in line with previous findings showing that epithelial TF exerts protective effects, e.g. supporting the alveolar–capillary barrier, in various infectious and non‐infectious ALI models 18, 35, 36. For instance, in an LPS‐induced lung inflammation model, the presence of epithelial cell TF determines the severity of the lung pathology 18, 33. Whereas, in LPS‐induced ALI, myeloid TF is of minor importance 36, we provide evidence that, in acid‐induced ALI, deficiency of myeloid TF promoted the inflammatory response and thereby significantly exacerbated disease progression. Increased inflammation may be the reason for increased TF expression in whole lung tissue, as observed in TF^Δmye^ mice as compared with wild‐type mice during ALI. Previous studies have indicated that the effect of myeloid TF depends significantly on the disease model. Whereas, in low‐TF mice, bacterial burden and cytokine release were unaltered as compared with wild‐type mice during tuberculosis 37, myeloid TF deficiency augmented the pulmonary growth of *Mycobacterium tuberculosis*, most likely because of reduced fibrin generation and shifting macrophage polarization 38.

Such disease model‐dependent differences could explain our observations regarding the role of myeloid TF in ALI. In contrast to LPS‐induced ALI, which induces apoptosis in epithelial and endothelial cells 39, 40, acid‐induced ALI creates a direct cell‐damaging disease pattern. Intratracheal instillation of HCl mainly affects epithelial cells, leading to impaired epithelial cell function and fluid transport, and indirect damage to the capillaries 41. Epithelial cell injury was also exemplified by damage‐associated release of IL‐33 into the bronchoalveolar space. IL‐33 is abundantly expressed in lung epithelial cells 42. However, we did not observe any differences in IL‐33 levels in BALF between TF^Δmye^ mice and wild‐type mice, indicating comparable initial insults by acid aspiration in both groups. However, during acid‐induced ALI, TF^Δmye^ mice showed increased pulmonary TF expression, indicating enhanced activation of non‐hematopoietic cells, e.g. type I and type II lung epithelial cells, upon acid injury. The most apparent differences in inflammation were significant increases in proinflammatory IL‐6 levels and leukocyte/neutrophil influx. These occurred independently of coagulation activation or the presence of TF on epithelial cells, and was solely dependent on myeloid TF. Although there was a tendency for there to be increased cytokine expression in TF^Δmye^ mice during LPS‐induced ALI, no significant differences were observed 36.

In line with elevated cytokine levels, we also observed increased recruitment of leukocytes into the bronchoalveolar space of TF^Δmye^ mice, comprising inflammatory macrophages and, particularly, neutrophils; however, total MPO levels were only slightly increased. These findings indicate that, although coagulation is not affected by the lack of TF on myeloid cells and, in particular, on alveolar macrophages, the proinflammatory environment created by acid‐induced ALI further substantiates the damage. This idea is supported by the significant upregulation of proinflammatory cytokines, e.g. IL‐6, by isolated TF‐deficient alveolar macrophages upon LPS stimulation *in vitro*.

However, neutrophils can also upregulate TF expression during inflammation 5, 43. Infection with influenza virus A/H1N1 induces TF expression on various cell types, including neutrophils 44. Thus, we cannot exclude the possibility that lack of TF on neutrophils contributes to the observed phenotype during ALI.

TF upregulation during ALI results not only in a mild hypercoagulable state but also in an inflammatory state, as FVIIa, FXa, and activated FII can activate protease‐activated receptors, which induce the expression of various cytokines 45. However, we provide evidence that myeloid TF itself dampens inflammatory reactions via downregulation of macrophage IL‐6 expression. Our data indicate that there is TF cell type‐specific and disease‐specific regulation of inflammatory responses, suggesting that TF acts as a double‐edged sword in the cross‐talk between inflammation and coagulation.

Taking our findings together, we conclude that myeloid TF has roles in addition to activating the extrinsic coagulation cascade, as TF clearly shows immunomodulatory potential. Thus, myeloid TF may potently regulate the intensity of inflammatory responses to exogenous lung insults such as that caused by gastric contents.

## Addendum

J. B. Kral‐Pointner designed and performed experiments, analyzed the results, and wrote the manuscript. W. C. Schrottmaier and V. Horvath planned and performed research, and analyzed the data. H. Datler was involved in data collection and animal experiments. C. Ay, B. Niederreiter, and L. Hell were involved in data collection. N. Mackman provided conditional knockout mice and supervised the study. B. Jilma and J. A. Schmid were involved in interpreting and discussing the results. A. Assinger and S. Knapp participated in study design and contributed to the discussion and interpretation of the data. G. Schabbauer designed the study, was involved in result interpretation and discussion of the research progress, and wrote the manuscript. All authors confirm that they had full access to the data and the manuscript; they revised its intellectual content and approved the final version.

## Disclosure of Conflict of Interests

The authors state that they have no conflict of interest.

## Supporting information


**Data S1.** Methods.
**Fig. S1.** Genotyping data of myeloid and airway epithelial TF‐deficient mice.
**Fig. S2.** Macrophage recruitment into the lung 8 h after acid‐induced acute lung injury (ALI).
**Fig. S3.** Myeloid TF does not influence leukocyte recruitment during sterile peritonitis.
**Fig. S4.** No significant changes in IL‐12 and IL‐1β levels between myeloid TF and wild‐type littermates 8 h after acid‐induced ALI.
**Fig. S5.** Effect of thrombin and FVIIa on the inflammatory response of macrophages.
