# Supplementary material for: Myeloid but not epithelial tissue factor exerts protective anti‐inflammatory effects in acid aspiration‐induced acute lung injury
Source: J Thromb Haemost. 2017 Jun 20;15(8):1625–39. doi: 10.1111/jth.13737 (PMC5575489; doi:10.1111/jth.13737)
Supplement: Supplementary file 1 — Data S1. Methods. Fig. S1. Genotyping data of myeloid and airway epithelial TF‐deficient mice. Fig. S2. Macrophage recruitment into the lung 8 h after acid‐induced acute lung injury (ALI). Fig. S3. Myeloid TF does not influence leukocyte recruitment during sterile peritonitis. Fig. S4. No significant changes in IL‐12 and IL‐1β levels between myeloid TF and wild‐type littermates 8 h after acid‐induced ALI. Fig. S5. Effect of thrombin and FVIIa on the inflammatory response of macrophages. [file JTH-15-1625-s001.pdf]

ONLINE DATA SUPPLEMENT

Myeloid but not epithelial tissue factor exerts protective anti-inflammatory properties in acid aspiration-induced acute lung injury

JB Kral-Pointner*, WC Schrottmaier*, V Horvath*, H Datler*, L Hell[†], B Niederreiter[‡], B Jilma[§], JA Schmid[¶], A Assinger*, N Mackman**, S Knapp[††, ‡‡,] and G Schabbauer*, [§§]

*Institute for Physiology, Center for Physiology and Pharmacology, Medical University of Vienna, Austria

[†] Clinical Division of Haematology and Haemostaseology, Department of Medicine I, Medical University of Vienna, Austria

[‡] Division of Rheumatology, Internal Medicine III, Medical University of Vienna, Austria.

[§]Departments of Clinical Pharmacology, Department of Medicine I, Medical University of Vienna, Austria

[¶]Department for Vascular Biology and Thrombosis Research, Center for Physiology and Pharmacology, Medical University of Vienna, Austria

**Division of Hematology/Oncology, Thrombosis and Hemostasis Program, UNC McAllister Heart Institute, University of North Carolina, Chapel Hill, USA

[††]CEMM, Research Center for Molecular Medicine of the Austrian Academy of Sciences, Vienna, Austria

[‡‡]Laboratory of Infection Biology,Department of Medicine I, Medical University of Vienna, Austria

Running head: Myeloid TF exerts anti-inflammatory effects in ALI

[§§]Address correspondence to:

Dr. Gernot Schabbauer
Centre for Physiology and Pathophysiology
Medical University of Vienna
Schwarzspanierstrasse 17
A-1090 Vienna, Austria
E-mail: gernot.schabbauer@meduniwien.ac.at
Phone: +43/1/40160/31427

Supplementary figures

Supplement figure I

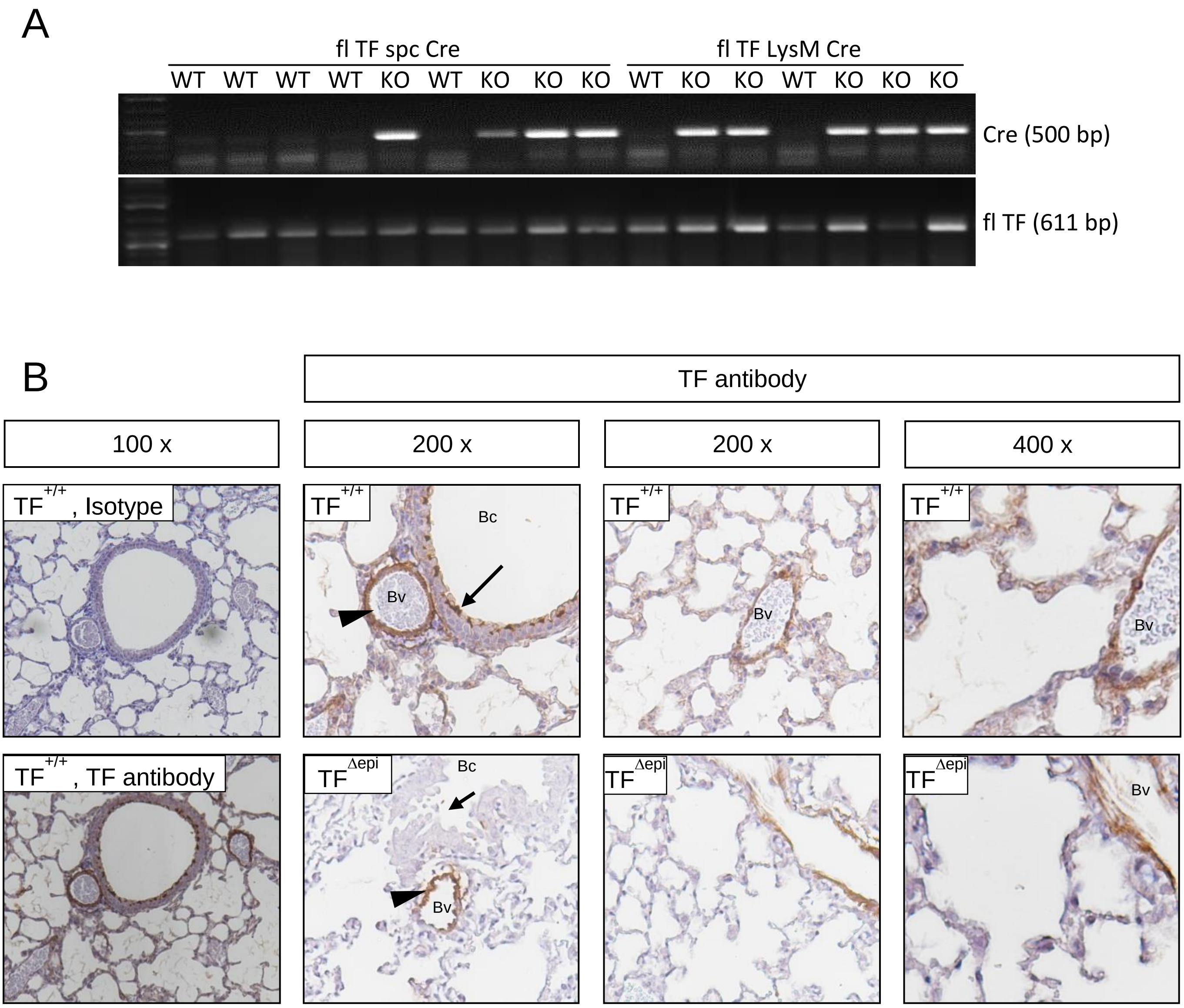

Supplement figure II

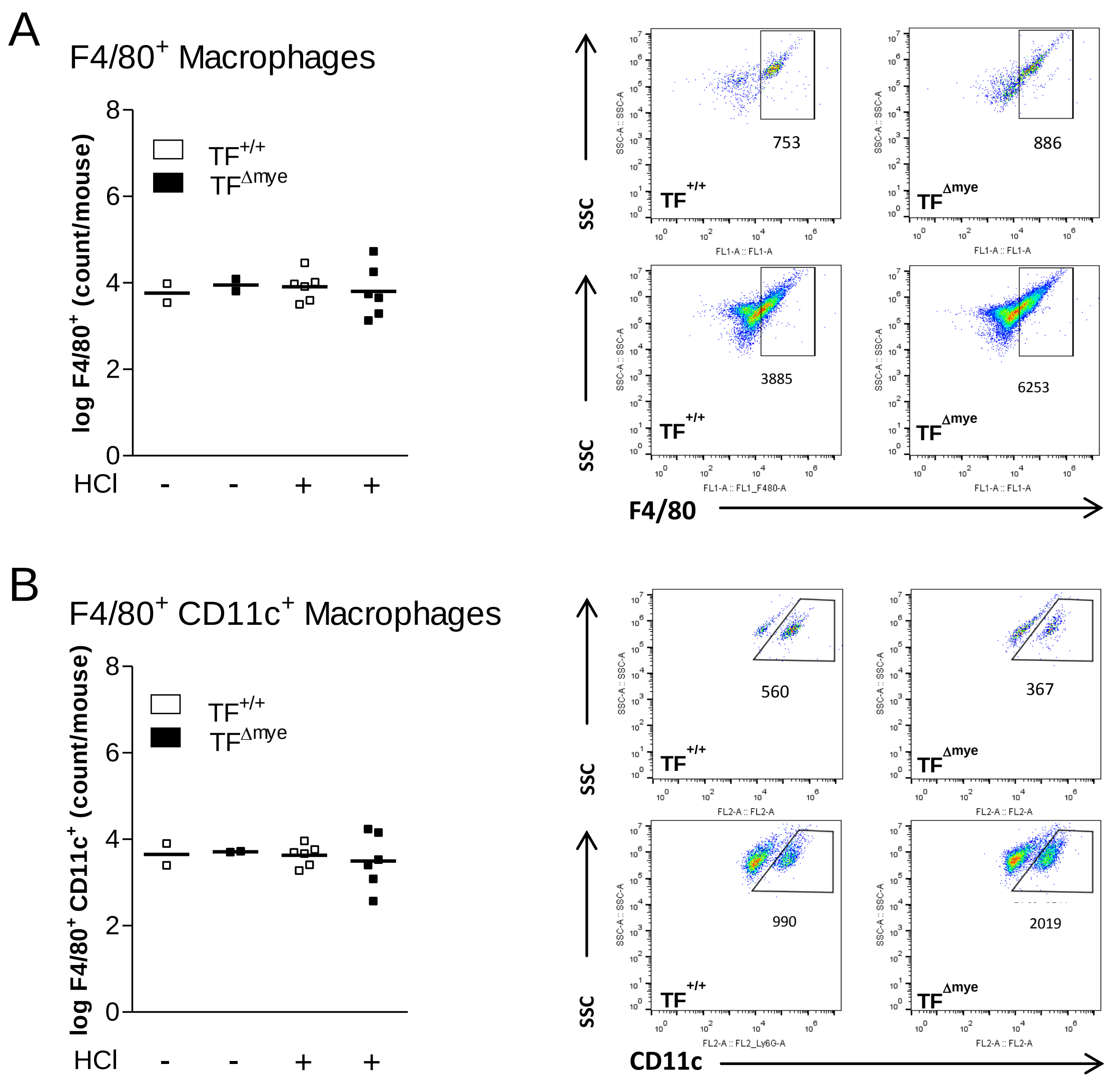

Supplement figure III

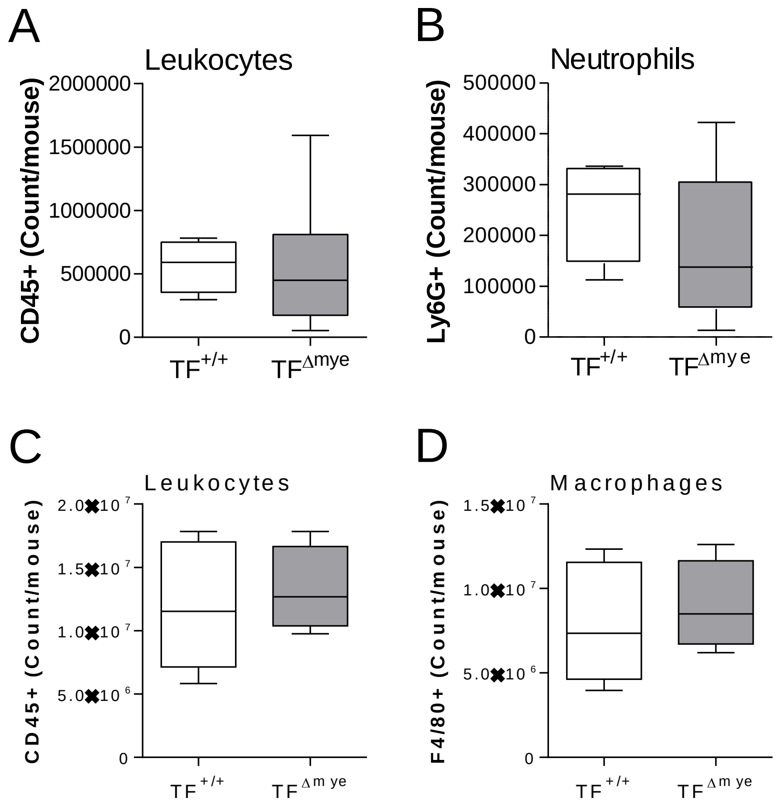

Supplement figure IV

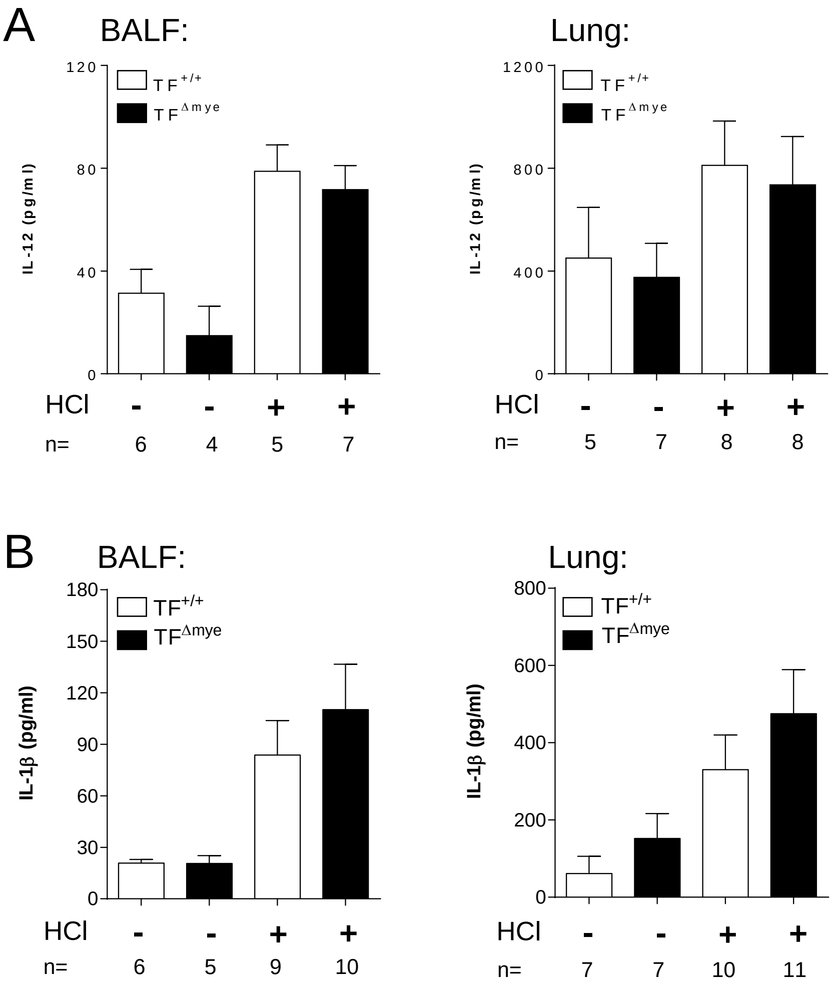

Supplement figure V

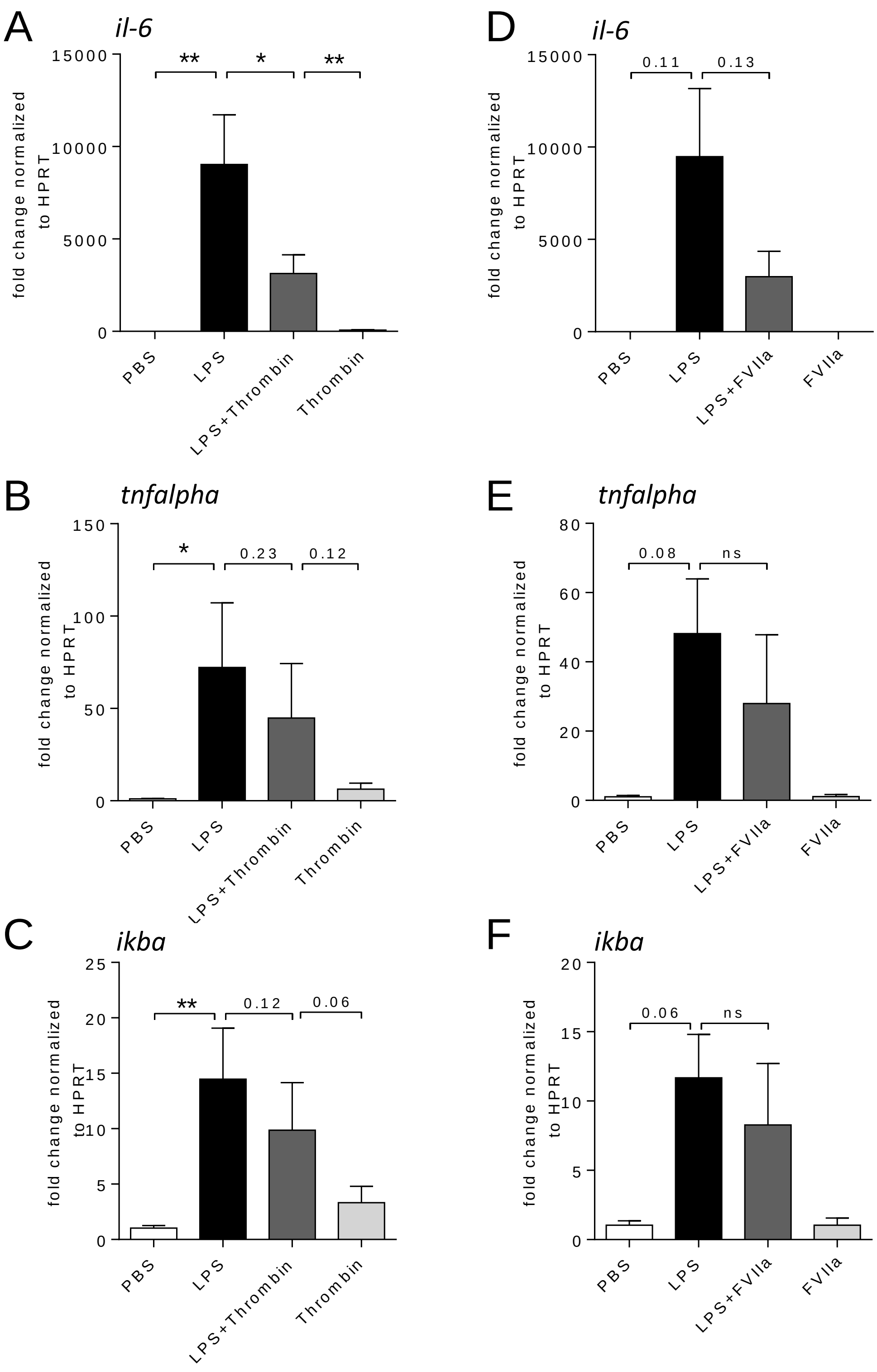

Supplementary figure legend

Supplemental Figure I: Genotyping data of myeloid and airway epithelial TF-deficient mice. (A) Analysis of genomic DNA of *TF* and *cre* alleles of tissue from $TF^{\Delta epi}$ and $TF^{\Delta mye}$ mice and respective $TF^{+/+}$ littermates by PCR was performed. (B) Histological assessment of naive lungs of wild-type ($TF^{+/+}$) and $TF^{\Delta epi}$ mice. First picture in upper panel shows isotype control staining and in the lower panel the TF antibody staining (brown). Second till fourth pictures shows $TF^{+/+}$ mice in the upper panel and $TF^{\Delta epi}$ mice in the lower panel stained for TF. Magnifications are from 100 x to 400 x as indicated, arrow head indicates endothelial cells, arrow indicates epithelial cells, blood vessel (Bv), bronchus (Bc).

Supplemental Figure II: Macrophage recruitment into the lung 8h post acid-induced acute lung injury (ALI). Accumulation of macrophage subpopulations ($CD45^+$ $F4/80^+$ (A) and $CD45^+$ $F4/80^+$ $CD11c^+$ (B)) in the broncho-alveolar lavage fluid of $TF^{+/+}$ and $TF^{\Delta mye}$ mice was analyzed by flow cytometry, $n_{control}$=2; n_{HCl}=6. (A, B) Representative flow cytometry blots were given. Upper panel control mice, lower level HCl-treated mice. For statistical analysis unpaired Student’s t-test was performed. Representative flow cytometry blots are given.

Supplemental Figure III: Myeloid TF does not influence leukocyte recruitment during sterile peritonitis. (A, B) Extravasation of leukocytes ($CD45^+$, A) and neutrophils ($CD45^+$ $Ly6G^+$ $F4/80^-$, B) was evaluated by flow cytometry 4 hours post thioglycollate intraperitoneal injection, $n_{TF+/+}$=4; $n_{TF\Delta mye}$=8. (C,D) Extravasation of leukocytes (C) and macrophages ($CD45^+$ $F4/80^+$, D) 72 hours post treatment, $n_{TF+/+}$=5; $n_{TF\Delta mye}$=4. For statistical analysis unpaired Student’s t-test was performed.

Supplemental Figure IV: No significant changes in IL-12 and IL-1β levels between myeloid TF and wildtype littermates 8h post acid-induced ALI. Concentrations of (A) IL-12 and (B) IL-1β in broncho-alveolar lavage fluid (BALF) or whole lung tissue were measured by ELISA. For statistical analysis unpaired Student’s t-test was performed.

Supplemental Figure V: Effect of thrombin and FVIIa on the inflammatory response of macrophages. Bone marrow cells were isolated and differentiated to bone marrow-derived macrophages by GM-CSF (10 ng/ml) and then stimulated with LPS (10 ng/ml), thrombin (0.66 U/ml) and FVIIa (8 ng/ml) for 3 hours as indicated. (A, D) IL-6, (B, E) TNF-α and (C, F) IκBα mRNA levels were determined by qRT-PCR and depicted as fold PBS control. One-way ANOVA with Tukey's multiple comparisons test was applied for statistical analysis; (A-C) n=5, (D-F) n=3. *$p<0.05$, **$p<0.01$, n.s. not significant.

Supplementary methods

Thioglycollate-elicited sterile inflammation

2 ml 4% thioglycollate medium (BD Becton, Dickinson and Company, New Jersey, USA) were injected intraperitoneally and after 4 hours or 72 hours a peritoneal lavage with 8ml PBS was performed. The recollected lavage fluid was centrifuged at 1000 xg for 5 min at room temperature and the cell pellet resupended in 0.5 ml PBS and stained for flow cytometry.

BMDM stimulation

BMDM isolation is described in the method section of the manuscript. BMDMs were stimulated with thrombin (0.66 U/ml, Technoclone, Vienna, Austria) and human FVIIa (8 ng/ml, Novo Nordisk, Vienna, Austria) for 3 hours.

Flow cytometry

Additionally used antibody: α-mouse CD11c-PE (1:80, BioLegend, London, UK).

qPCR

The following primers were applied: HPRT forward: 5'-CGCAGTCCCAGCGTCGTG-3', reverse: 5'-CCATCTCCTTCATGACATCTCGAG-3'; IL-6 forward: 5'-CAAGTCGGAGGCTTAATTACACATG-3', reverse:5'-ATTGCCATTGCACAACTCTTTTCT-3'; TF forward: 5'-CAGTTCATGGAGACGGAGAC-3', reverse: 5'-CAACCACGTTCAGTTTTCTACC-3'; TNF-α forward: 5'- CCACCACGCTCTTCTGTCTAC-3', reverse: 5'- AGGGTCTGGGCCATAGAACT-3'; EGR1 forward: 5'-AGCGAACAACCCTATGAGC-3', reverse: 5'- AGGCCACTGACTAGGCTGAA-3'; IκBα forward: 5'- GAAGCCGCTGACCATGGAA-3', reverse: 5'-GATCACAGCCAAGTGGAGTGGA-3', STAT1 forward: 5'- GCTGCCTATGATGTCTCGTTT-3', reverse: 5'- TGGACATCTGTACGGGATCTT-3'
